# A three-dimensional geometric morphometric analysis of the morphological transformation of *Caiman* lower jaw during post-hatching ontogeny

**DOI:** 10.7717/peerj.15548

**Published:** 2023-07-12

**Authors:** María Victoria Fernandez Blanco, Guillermo Hernán Cassini, Paula Bona

**Affiliations:** 1 División Paleontología Vertebrados, Museo de La Plata, Unidades de Investigación Anexo II Museo, Facultad de Ciencias Naturales y Museo, Universidad Nacional de La Plata, La Plata, Buenos Aires, Argentina; 2 Consejo Nacional de Investigaciones Científicas y Técnicas (CONICET), Ciudad Autónoma de Buenos Aires, Argentina; 3 División Mastozoología, Museo Argentino de Ciencias Naturales “Bernardino Rivadavia”, Ciudad Autónoma de Buenos Aires, Argentina; 4 Departamento de Ciencias Básicas, Universidad Nacional de Luján, Luján, Buenos Aires, Argentina

**Keywords:** Crocodylia, Feeding ecology, Mandible, Shape changes

## Abstract

Shape ontogenetic changes of the lower jaw in crocodylians are poorly understood. In order to answer some questions related to the inter- and intraspecific morphological variation of the mandible of two extant *Caiman* species, we performed a three-dimensional geometric morphometric approach. For this purpose, we used landmarks and semilandmarks on two ontogenetic mandibular series of 48 and 15 post-hatching specimens of *C. yacare* and *C. latirostris*, respectively. We have also examined the relationship between these anatomical transformations and ontogenetic shifts in diet. We performed a principal component analysis (PCA) for the two species, and regression and partial least squares (PLS) analyses for each species, separately. As a result, species were segregated along the PC1 with specimens of *C. yacare* showing more gracile mandibles, and specimens of *C. latirostris* more robust ones. The PC2 and regression analyses showed an age gradient and represented ontogenetic shape changes. Adult caiman mandibles are higher and wider than juvenile ones, and shape changes are more conspicuous in *C. latirostris*. The PLS analyses showed a significant relationship between shape and diet. Morphological changes of the PLS1 of block-1 match with those of the regression analysis for both species. We have detected morphological transformations in areas where the musculature in charge of mandibular movements is attached. Common morphological changes occurring during ontogeny seem to reflect the same mechanical properties required for crushing and killing in both species, driven by an ontogenetic shift in the diet from invertebrates to vertebrates. Additionally, interspecific differences were also found to be correlated to ontogenetic changes in diet and could be related to dissimilar feeding mechanical requirements (*e.g*., stiffness and toughness of the item consumed), and to different habitat preferences. Robust mandibles would be more suitable for shallow and fully vegetated environments, as it can be seen in *C. latirostris*, whereas slender jaws seem to be more suitable for more aquatic species such as *C. yacare*.

## Introduction

Crocodylia is a clade represented by extinct and extant species that has evolved a huge spectrum of cranial morphological variation since its origin in the Late Cretaceous (*e.g*., Brochu, 1999, 2001, 2003; Jouve et al., 2008; Blanco et al., 2015; Bronzati, Montefeltro & Langer, 2015; Rio & Mannion, 2021). The group has a long and rich fossil record, spanning a wide range of skull shapes and body sizes (*e.g*., Gearty & Payne, 2020; Rio & Mannion, 2021; Stockdale & Benton, 2021; Stocker, Brochu & Kirk, 2021). Although extinct crocodylians vastly outnumber their modern relatives and the current morphological diversity is lower compared to that of the past (*e.g*., Brochu, 2001, 2003; Bronzati, Montefeltro & Langer, 2015; Mannion et al., 2015; Godoy, 2020; Rio & Mannion, 2021), extant species still exhibit a considerable variety of cranial shapes (Pierce, Angielczyk & Rayfield, 2008). In this sense, skull disparity has attracted the attention of researchers for many years, becoming the focus of several studies (*e.g*., Brochu, 1999, 2001; Busbey, 1995; McHenry et al., 2006; Pierce, Angielczyk & Rayfield, 2008, 2009a, 2009b; Piras et al., 2010, 2014; Pearcy & Wijtten, 2011; Fernandez Blanco, Cassini & Bona, 2014; Fernandez Blanco et al., 2015; Fernandez Blanco, Cassini & Bona, 2018; Escobedo-Galvan et al., 2015; Foth et al., 2017; Angulo-Bedoya, Correa & Benítez, 2019; Morris et al., 2019, 2021; Falcón Espitia & Jerez, 2021). It is not only the peculiar head configuration that makes the skull so broadly studied but also its greater presence in the fossil record and museum collections, compared to other components of the skeleton. Furthermore, the comparative study of the crocodylian skull is crucial since cranial features are essential for phylogenetic analyses, and therefore, for the evolutionary history of the group (*e.g*., Brochu, 1997, 1999, 2011; Hua & Jouve, 2004; Aguilera, Riff & Bocquentin-Villanueva, 2006; Bona, 2007; Jouve et al., 2008, 2015; Brochu & Storrs, 2012; Hastings et al., 2013; Hastings, Reisser & Scheyer, 2016; Scheyer et al., 2013; Fortier et al., 2014; Salas-Gismondi et al., 2015, 2016; Cidade et al., 2017; Massonne et al., 2019; Souza-Filho et al., 2019; Ristevski et al., 2021; Rio & Mannion, 2021).

Exploring the literature regarding crocodylian skull morphologies, several quantitative studies examine the patterns of cranial variation and their biomechanical, functional, and ecological implications (*e.g*., Iordansky, 1973; Busbey, 1989; Daniel & McHenry, 2001; McHenry et al., 2006; Pierce, Angielczyk & Rayfield, 2008, 2009a, 2009b; Sadleir & Makovicky, 2008; Piras et al., 2009, 2014; Erickson et al., 2012; Walmsley et al., 2013; Fernandez Blanco, Cassini & Bona, 2014; Fernandez Blanco et al., 2015; Fernandez Blanco, Cassini & Bona, 2018; Gignac & Erickson, 2016; McCurry et al., 2017; Ballell et al., 2019; Felice, Pol & Goswami, 2021). In all these articles, different ontogenetic stages after hatching of extinct and/or modern species are analyzed. However, it is worth pointing out that these researchers have paid attention only to the cranium and have neglected or only partially analyzed the lower jaw. This is striking since mandibles comprise meaningful elements to varying biological and paleobiological approaches (*e.g*., morphofunctional studies and feeding ecology; see Vizcaíno et al., 2016) as they may provide much anatomical information that can be extracted from bone correlates (such as crests and marks of muscle attachments, and nerves or blood vessels canals and foramina) (*e.g*., Iordansky, 1964, 2000, 2010; Schumacher, 1973, 1985; Bona & Desojo, 2011; Porro et al., 2011; Stubbs et al., 2013; Walmsley et al., 2013). Furthermore, mandibles are easy-to-handle elements (to measure and digitalize) in museum collections due to their sizes, and their study is not relatively hard since they are simpler structures than crania because they are made up of fewer elements. Despite this, there are few works dealing with crocodylian mandibles. Monteiro & Soares (1997) and Verdade (2000) analyzed the ontogenetic variation of the skull shape of three *Caiman* species (*C. latirostris* (Daudin, 1802), *C. yacare* (Daudin, 1802) and *C. sclerops* Schneider, 1801), and included few linear measurements of the lower jaw. Escobedo-Galvan et al. (2015) also used conventional metrics of the skull and mandibles, and evaluated the morphological position of *C. crocodilus apaporiensis* within the morphospace of *C. crocodilus* complex and *C. yacare* (ontogeny was not examined). Confirming and complementing the results of the latter, Angulo-Bedoya, Correa & Benítez (2019) applied a two-dimensional geometric morphometric approach over cranial and mandibular series of this *C. crocodilus* subspecies complex. On the other hand, Porro et al. (2011), Stubbs et al. (2013) and Walmsley et al. (2013) made the first comprehensive biomechanical analyses focused exclusively on different crocodylian mandibular metrics, and analyzed their relation to ecological diversity and feeding/mechanical behaviors. In sum, beyond these articles, there is no other study that merely emphasizes the quantitative analysis of the ontogenetic morphological transformation of the lower jaw in crocodylians or its morphological interspecific variation.

Caimaninae is an alligatoroid crocodylian clade currently restricted to South and Central America (Simpson, 1937; Gasparini, 1996; Brochu, 1999, 2010, 2011; Bona, 2007; Pinheiro et al., 2012; Salas-Gismondi et al., 2015). Its fossil record is usually fragmentary and species diagnosis are mostly built over characters extracted from cranial and mandibular materials which are generally broken, incomplete, and/or deformed (*e.g*., Brochu, 1999, 2011; Aguilera, Riff & Bocquentin-Villanueva, 2006; Bona, 2007; Hastings et al., 2013; Hastings, Reisser & Scheyer, 2016; Scheyer et al., 2013; Fortier et al., 2014; Salas-Gismondi et al., 2015, 2016; Hastings, Reisser & Scheyer, 2016; Cidade et al., 2017; Souza-Filho et al., 2019; Rio & Mannion, 2021). Furthermore, some extinct caimanine species were identified and described only based on mandibular fragments (*e.g*., *Eocaiman palaeocenicus*: Bona, 2007, *E. itaboraiensis*: Pinheiro et al., 2012, *Caiman tremembensis:* Chiappe, 1988). Since limits currently used to delineate fossil species are based on ranges of variation of modern specimens (Brochu & Sumrall, 2020), any study analyzing cranial and lower jaw morphological variation of extant species (such as morphological transformations during ontogeny) would help us more accurately identify characters states used in systematics. In this sense, Fernandez Blanco, Cassini & Bona (2014, 2018) made the first morphogeometric contribution and quantified the ontogenetic morphological variation in the skull of two extant *Caiman* species, identifying cranial features that vary along ontogeny and are relevant in systematics. Given that Caimaninae is a clade that holds extant relatives, it comprises an excellent opportunity to study the development of bone features and to extrapolate this information with fossil specimens in order to reinforce paleobiological and paleontological taxonomic hypotheses.

The aim of this study is to analyze the ontogenetic morphological transformations that undergo the mandibles of two extant caimanine species, *C. latirostris* and *C. yacare*, and their interspecific variation, applying a three-dimensional geometric morphometric approach. Special attention will be paid to: 1-common patterns of ontogenetic change in both *Caiman* species and; 2-morphological features that remain stable during the post-hatching ontogeny. Given the principal role of jaws in food intake, we will also assess the degree to which diet and morphological disparity covary. Feeding ecology across different age categories of these crocodylian species has been analyzed in some previous quantitative studies such as Borteiro et al. (2008), Santos et al. (1996) and Fernandez Blanco, Cassini & Bona (2018). Although *C. latirostris* is considered as a more durophage species than *C. yacare*, juveniles of both species feed mainly on invertebrates such as insects, snails, crustaceans and spiders, and adults feed mainly on vertebrates. However, even though the size of the prey consumed by the two *Caiman* species increases throughout development as well as their frequency of consumption, predation on small prey never ceases (Santos et al., 1996; Melo, 2002; Borteiro et al., 2008). Thus, finally, we discuss about the relationship between the morphological variation of these crocodylian species to their habitat and feeding ecology.

## Materials and Methods

We examined two post-hatching ontogenetic series of mandibles of two modern species of *Caiman* that inhabit Argentina, *C. latirostris* and *C. yacare*. Samples belonged to wild animals coming mostly from Argentina (Chaco and Corrientes Provinces; Table 1) and sex, age, and sexual or somatic maturity are not determined. The material is housed at the Museo de La Plata (MLP) and Museo Argentino de Ciencias Naturales “Bernardino Rivadavia” (MACN).

**Table 1:** List of material used for the analysis with collection’s number of each specimen, repository, provenance and ontogenetic stage.

Species	Collection number	Procedence	Ontogenetic stage
*Caiman latirostris*	MLP-R.5801	Chaco Province, Argentina	Juvenile
*Caiman latirostris*	MLP-R.5803	Chaco Province, Argentina	Juvenile
*Caiman latirostris*	MLP-R.5808	Chaco Province, Argentina	Juvenile
*Caiman latirostris*	MACN-30565	Corrientes Province, Argentina	Subadult
*Caiman latirostris*	MLP-R.5811	Chaco Province, Argentina	Subadult
*Caiman latirostris*	MACN-30567	Corrientes Province, Argentina	Subadult
*Caiman latirostris*	MACN-30572	Corrientes Province, Argentina	Subadult
*Caiman latirostris*	MLP-R.5809	Chaco Province, Argentina	Subadult
*Caiman latirostris*	MLP-R.5810	Chaco Province, Argentina	Subadult
*Caiman latirostris*	MACN-30566	Corrientes Province, Argentina	Adult
*Caiman latirostris*	MLP-R.5364	Chaco Province, Argentina	Adult
*Caiman latirostris*	MACN-30610	Corrientes Province, Argentina	Adult
*Caiman latirostris*	MLP-R.5038	Chaco Province, Argentina	Adult
*Caiman latirostris*	MLP-R.5043	Corrientes Province, Argentina	Adult
*Caiman latirostris*	MLP-R.6251	Corrientes Province, Argentina	Adult
*Caiman yacare*	MACN-30590	Corrientes Province, Argentina	Juvenile
*Caiman yacare*	MACN-30635	Corrientes Province, Argentina	Juvenile
*Caiman yacare*	MLP-R.5048	Corrientes Province, Argentina	Juvenile
*Caiman yacare*	MLP-R.5049	Corrientes Province, Argentina	Juvenile
*Caiman yacare*	MLP-R.5052	Corrientes Province, Argentina	Juvenile
*Caiman yacare*	MLP-R.5053	Corrientes Province, Argentina	Juvenile
*Caiman yacare*	MLP-R.5057	Corrientes Province, Argentina	Juvenile
*Caiman yacare*	MACN-30527	Corrientes Province, Argentina	Subadult
*Caiman yacare*	MACN-30533	Corrientes Province, Argentina	Subadult
*Caiman yacare*	MACN-30583	Corrientes Province, Argentina	Subadult
*Caiman yacare*	MACN-30584	Corrientes Province, Argentina	Subadult
*Caiman yacare*	MACN-30631	Corrientes Province, Argentina	Subadult
*Caiman yacare*	MLP-R.5040	Corrientes Province, Argentina	Subadult
*Caiman yacare*	MACN-30534	Corrientes Province, Argentina	Subadult
*Caiman yacare*	MACN-30537	Corrientes Province, Argentina	Subadult
*Caiman yacare*	MACN-30540	Corrientes Province, Argentina	Subadult
*Caiman yacare*	MACN-30561	Corrientes Province, Argentina	Subadult
*Caiman yacare*	MACN-30562	Corrientes Province, Argentina	Subadult
*Caiman yacare*	MACN-30563	Corrientes Province, Argentina	Subadult
*Caiman yacare*	MACN-30593	Corrientes Province, Argentina	Subadult
*Caiman yacare*	MACN-30599	Corrientes Province, Argentina	Subadult
*Caiman yacare*	MACN-30607	Corrientes Province, Argentina	Subadult
*Caiman yacare*	MACN-30625	Corrientes Province, Argentina	Subadult
*Caiman yacare*	MACN-30626	Corrientes Province, Argentina	Subadult
*Caiman yacare*	MLP-R.5805	Chaco Province, Argentina	Subadult
*Caiman yacare*	MACN-I.15144	Chaco Province, Argentina	Adult
*Caiman yacare*	MACN-30539	Corrientes Province, Argentina	Adult
*Caiman yacare*	MACN-30541	Corrientes Province, Argentina	Adult
*Caiman yacare*	MACN-30542	Corrientes Province, Argentina	Adult
*Caiman yacare*	MACN-30544	Corrientes Province, Argentina	Adult
*Caiman yacare*	MACN-30548	Corrientes Province, Argentina	Adult
*Caiman yacare*	MACN-30550	Corrientes Province, Argentina	Adult
*Caiman yacare*	MACN-30560	Corrientes Province, Argentina	Adult
*Caiman yacare*	MACN-30574	Corrientes Province, Argentina	Adult
*Caiman yacare*	MACN-30637	Corrientes Province, Argentina	Adult
*Caiman yacare*	MACN-30638	Corrientes Province, Argentina	Adult
*Caiman yacare*	MACN-I8266	Paraguay	Adult
*Caiman yacare*	MACN-I8268	Paraguay	Adult
*Caiman yacare*	MLP-R.5041	Corrientes Province, Argentina	Adult
*Caiman yacare*	MLP-R.5042	Corrientes Province, Argentina	Adult
*Caiman yacare*	MLP-R.5044	Corrientes Province, Argentina	Adult
*Caiman yacare*	MLP-R.5045	Chaco Province, Argentina	Adult
*Caiman yacare*	MACN-30536	Corrientes Province, Argentina	Adult
*Caiman yacare*	MACN-30543	Corrientes Province, Argentina	Adult
*Caiman yacare*	MACN-30553	Corrientes Province, Argentina	Adult
*Caiman yacare*	MACN-30595	Corrientes Province, Argentina	Adult
*Caiman yacare*	MACN-30602	Corrientes Province, Argentina	Adult
*Caiman yacare*	MACN-I8267	Paraguay	Adult

**Figure 1:**
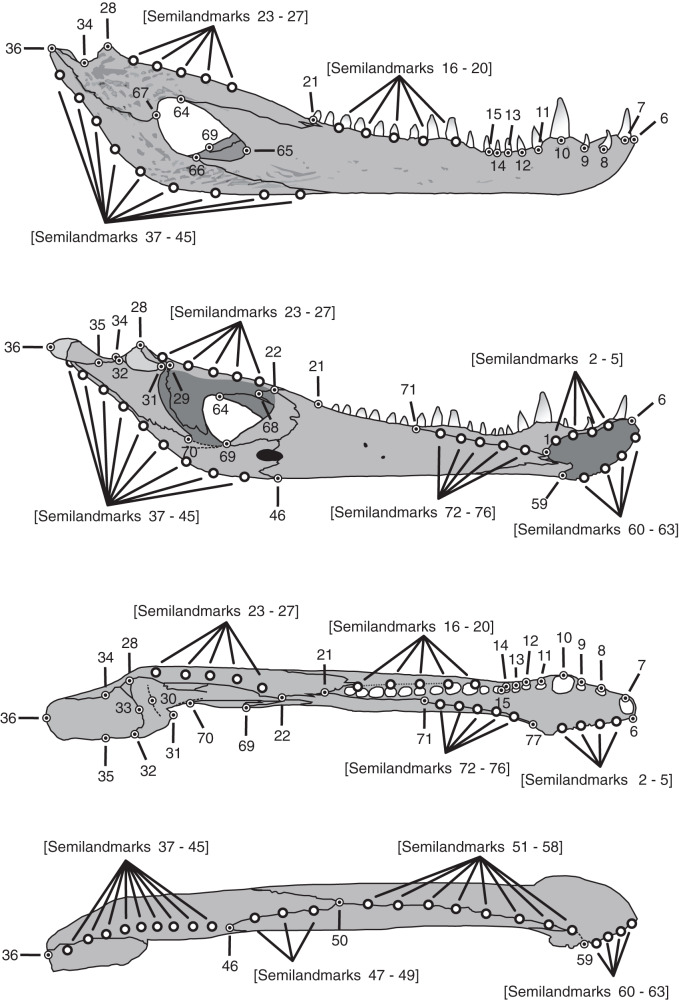
Landmarks and semilandmarks chosen to depict morphological change in the *Caiman* mandibles. In the figure, from top to bottom, the views are: lateral, medial, dorsal and ventral.

A three-dimensional geometric morphometric approach was implemented to evaluate and visualize the morphological transformations that mandibles undergo from juvenile to adult individuals. For this purpose, jaws were assigned to three different age categories (juveniles, subadults and adults) following the same criteria used in Fernandez Blanco, Cassini & Bona (2018) (Table 1), which defined these classes by the snout-vent length (see Santos et al. (1996) and Borteiro et al. (2008)). The *C. latirostirs* sample consisted of three juveniles, six subadults and six adults, whereas the *C. yacare* sample consisted of seven juveniles, 18 subadults and 23 adults. A total of 77 landmarks (Type I, II, and semilandmarks; Fig. 1; Table 2) were digitized on 63 jaws (48 jaws of *C. yacare* and 15 jaws of *C. latirostris*) using a Microscribe G2L digitizer.

**Table 2:** Landmarks and semilandmarks used in this study.

Number	Anatomical description of landmarks and semilandmarks
1	Dorsal and posterior point of the mandibular symphysis
2–5	Semilandmarks between LM1 and LM6 along the dorsal margin of the mandibular symphysis
6	Dorsal and anterior point of the mandibular symphysis
7	Point on the external alveolar margin of tooth 1
8	Point on the external alveolar margin of tooth 2
9	Point on the external alveolar margin of tooth 3
10	Point on the external alveolar margin of tooth 4
11	Point on the external alveolar margin of tooth 5
12	Point on the external alveolar margin of tooth 6
13	Point on the external alveolar margin of tooth 7
14	Point on the external alveolar margin of tooth 8
15	Anterior point of the alveolar canal
16–20	Semilandmarks between LM15 and LM21 along the external alveolar margin of the alveolar canal or along the alveolar margin of teeth when the alveolar canal is absent.
21	Posterior point of the alveolar canal
22	Contact among splenial, coronoid and surangular
23–27	Semilandmarks between LM22 and LM28 along the dorsal margin of the surangular
28	Most posterdorsal point of contact between surangular and articular on the glenoid cavity
29	Most anterodorsal point of contact between surangular and articular on the glenoid cavity
30	Middle point of the anterior concavity of the glenoid cavity
31	Most anterior and medial point of the glenoid cavity
32	Most posterior and medial point of the glenoid cavity
33	Middle point of the posterior concavity of the glenoid cavity
34	Point on the concavity over the contact between articular and surangular
35	Projection of LM34 onto the medial border of the retroarticular process
36	Most posterior point of the articular
37–45	Semilandmarks between LM36 and LM46 along the ventral margin of the articular and angular
46	Most posterior contact between splenial and angular
47–49	Semilandmarks between LM46 and LM50 along the ventral margin of the jaw in the contact between splenial and angular
50	Contact among splenial, angular and dentary
51–58	Semilandmarks between LM50 and LM59 along the ventral margin of the jaw in the contact between dentary and splenial, and ventral margin of the jaw in the dentary
59	Ventral and posterior point of the mandibular symphysis
60–63	Semilandmarks between LM6 and LM59 along the ventral margin of the mandibular symphysis
64	Contact between dentary and surangular in the external mandibular fenestra
65	Anterior point of the external mandibular fenestra in the dentary
66	Contact between dentary and angular in the external mandibular fenestra
67	Contact between angular and surangular in the external mandibular fenestra
68	Most posterior contact between coronoid and surangular
69	Contact between angular and coronoid in the internal mandibular fenestra
70	Contact between articular and angular in the internal mandibular fenestra
71	Posterior contact between splenial and dentary on the dorsal internal margin
72–76	Semilandmarks between LM71 and LM77 along the dorsal contact between dentary and splenial
77	Most anterior and dorsal border of the splenial and dentary contact

There are 20/48 specimens of *C. yacare* with missing landmarks. Fourteen of them have less than five missing landmarks and six have more than seven. The regions of the mandible more frequently broken include the bone contacts in the external and internal mandibular fenestrae, coronoid, medial glenoid cavity, and less frequently, the retroarticular process. In *C. latirostris* 9/15 specimens have missing landmarks. Five of them have less than six missing landmarks, and four have more than eight. The regions of the mandible more frequently broken in this species include the bone contacts in the internal mandibular fenestrae, coronoid and the retroarticular process. The missing landmarks in broken mandibles were estimated to maximize the number of specimens in the sample. For a detailed procedure, see Fernandez Blanco, Cassini & Bona (2018) and the Supplemental Material 1 therein. Semilandmarks were equispaced using the Botton-Divet et al. (2016) Supplemental 2 for curve re-sampling script, and then landmarks configurations were superimposed using a generalized procrustes analysis (GPA) to remove the effects of orientation, positioning, and scaling. A principal component analysis (PCA) was performed to explore morphological changes. These analyses were implemented in *procSym* function of Morpho v2.5.1 R package (Schlager, 2017). The method proposed by Bookstein (2014) implemented in the function *getMeaningfulPCs* from same package was applied to determine whether a PC is eligible to interpret. In addition, Pearson correlation tests were performed to assess the extent and significance of the association between centroid size and pc scores.

Ontogenetic allometry deals with covariation among metric characters and corresponds to shape changes during growth of an individual (Klingenberg, 1996). Allometry could be expressed at the interspecific level (*e.g*., functional changes from an evolutionary perspective) or at the intraspecific level (*e.g*., allometry of growth) (see Klingenberg & Zimmermann, 1992; Klingenberg, 1996; Cassini, Flores & Vizcaíno, 2015 and references there in). In the GPA, shape and size were decomposed where centroid size was stored and used as a proxy for size (Goodall, 1991; Dryden & Mardia, 1998). Size was removed during the GPA, but the allometric component was not (Mitteroecker et al., 2013). In the absence of allometry, centroid size does not correlate with shape (Bookstein, 1986; Kendall, 1986). Therefore those components of shape that increase in size without correlation with size are isometric. To explore how shape variation is associated with size, and shape describes the relative size increase or decrease of specific parts (see Segura et al., 2017), we performed a multivariate regression (*lm* base function and *RegScore* from Morpho; see Schlager, 2022) on the Procrustes coordinates against the log-transformed centroid size for each species, separately (Klingenberg, 2016). Then the predicted component was expressed as a percentage of the total variation (*i.e*., coefficient of determination, *R*^2^) to quantify the shape variation explained by size in the dataset (Drake & Klingenberg, 2008; Cassini, 2013; Segura, Prevosti & Cassini, 2013; Segura et al., 2017). We also used the two-block partial least squares analysis (PLS: *pls2B* function from Morpho) to explore the patterns of covariation between mandibular morphology and diet through ontogeny of both caiman species (Mitteroecker & Bookstein, 2008). All information regarding feeding ecology was obtained from literature and analyzed following the same criteria used in Fernandez Blanco, Cassini & Bona (2018). We defined Block-1 as mandibular shape (*i.e*., landmark configurations) and Block-2 as continuous diet characters (*i.e*., the logit transformed diet proportions matrix; see Olsen, 2017; Fernandez Blanco, Cassini & Bona, 2018; Cassini & Toledo, 2021). PCA, PLS and Regressions produce vectors in shape space in different directions which were compared (angular comparison; see Segura, Prevosti & Cassini, 2013; Cassini, Muñoz & Vizcaino, 2017; Segura et al., 2017). The angles between these vectors were computed and compared using the *angleTest* function of Morpho package (see Li, 2011). When these angles are close to zero, this means that both analyses are similar, and consequently, share a similar shape change (Drake & Klingenberg, 2008; Klingenberg & Marugán-Lobón, 2013). All analyses were performed using the R v4.2.2 environment (R Core Team, 2022). The visualization and graphics to see the colour pattern associated with shape changes were made following Muñoz et al. (2017) and using the Morpho R package 2.5.1 (Schlager, 2017). Four specimens (one juvenile and adult species) were chosen and used to design the template average mesh for shape change visualizations. Landmarks and semi-landmarks were manually added to these meshes in the Landmark Editor. Semilandmarks were equispaced as it was described above. In each analysis, the corresponding meshes were transformed to consensus and then to the target shape (*tps3d* function) to finally compute a colored mesh representing the distances (as percent of centroid size) between the vertices of target and consensus meshes. The color keys are interpreted as follows: gray means no relative changes; red means relative increase in size and blue relative decrease in size. Additionally, a slide of thin plate spline gridlines was computed using the *deformGrid3d* function.

Raw data, meshes and supporting information are supplied on CONICET institutional repository (https://ri.conicet.gov.ar/handle/11336/184901).

## Results

The PCA resulted in ten principal components that account for the total cumulative variance. Accordingly to the *getMeaningfulPCs* function report, only the two first axes were meaningful and used to describe the total variation. The PC1 explains the 33.08% of the total variance whereas PC2 describes the 16.36%. Only PC2 correlates significantly and positively with log-transformed centroid size (*r* = 0.6546, *p*-value < 0.0001) and has a vector angle of 34.544° with the pooled within-group (species) regression of shape coordinates against log-transformed centroid size (*p*-value < 0.00001). That means that morphological changes along the PC2 are mostly associated with an age gradient, and partially represent the shared shape variation explained by size (see below).

**Figure 2:**
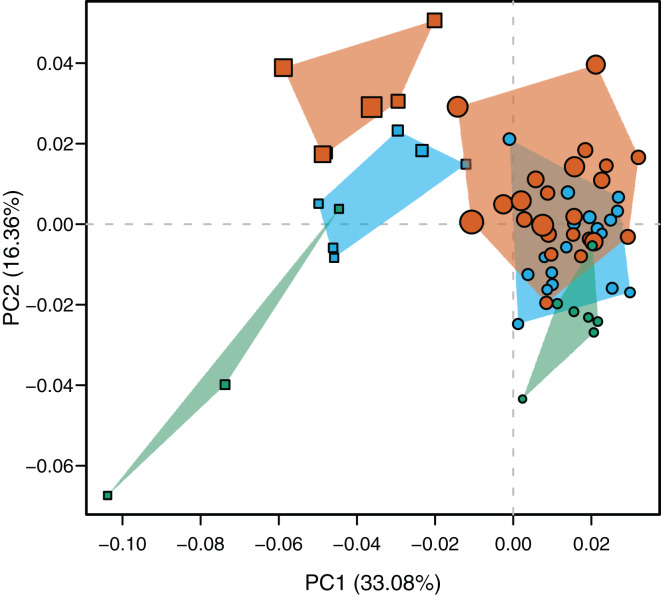
Scatterplot of morphospace depicted by PC1 *vs* PC2. *Caiman yacare* (circles) and *C. latirostris* (squares). Juveniles (green), sub-adults (blue), adults (red).

**Figure 3:**
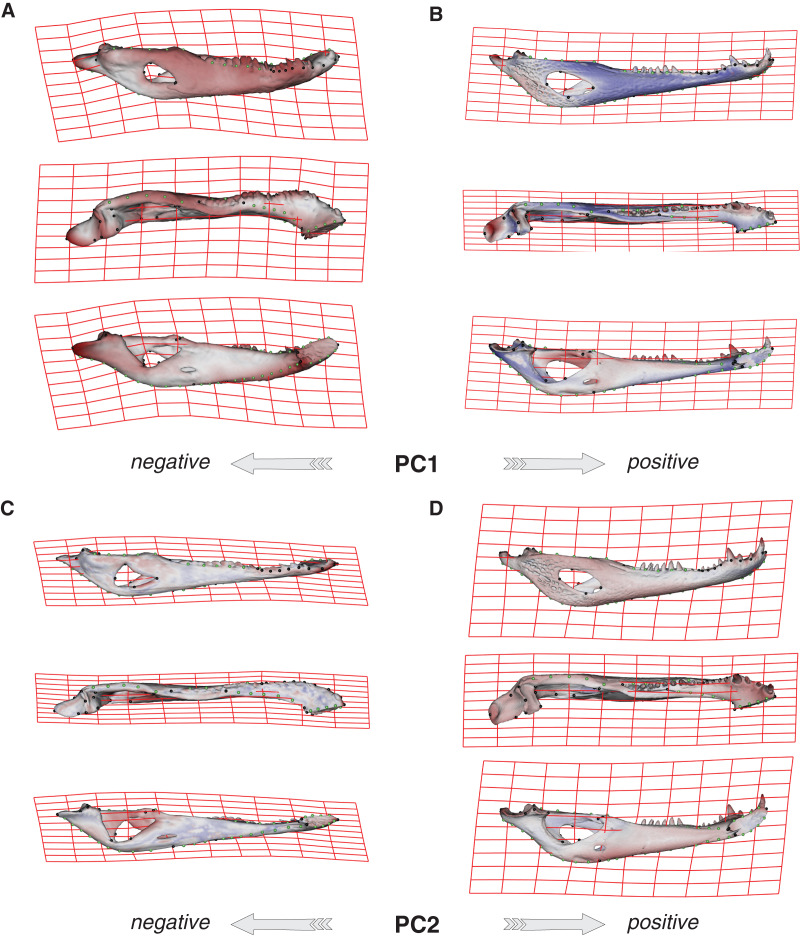
Thin plate spline gridlines, coloured meshes and shape changes associated with the PC1 and PC2. (A) Thin plate spline gridlines and coloured meshes of negative shape scores of PC1. (B) Thin plate spline gridlines and coloured meshes of positive shape scores of PC1. (C) Thin plate spline gridlines and coloured meshes of negative shape scores of PC2. (D) Thin plate spline gridlines and coloured meshes of positive shape scores of PC2. Colour intensity is proportional to the amount of change.

Species were segregated along the PC1 with *C. yacare* located mainly on the positive values (from −0.01 to 0.03 approximately) and *C. latirostris* on the negative ones (from −0.10 to −0.01) (Fig. 2). Both species slightly overlie near the value of −0.01 of the PC1. Shape changes associated with negative values of PC1 shows tall and wide mandibles (essentially in medial section) with small internal and external mandibular fenestrae, a large Meckelian fossa (area between both fenestrae), a dorsally curved dorsal area of the surangular bone, a massive articular bone with a wide articular area and a postero-ventrally inclined retroarticular process, a high and straight symphyseal area, not procumbent teeth, and a curved postero-ventral area of the angular bone (Fig. 3A). Instead, positive values show low and narrow mandibles (essentially in medial section) with large internal and external mandibular fenestrae, a small Meckelian fossa, a straight dorsal area of the surangular bone, a slender articular bone with a narrow articular area and a horizontal retroarticular process, a low and curved symphyseal area, procumbent teeth, and a straight postero-ventral area of the angular bone (Fig. 3B).

Age categories of *C. latirostris* were segregated along the PC2 with juveniles and adults lying on the extreme negative and positive values, respectively (Fig. 2). The three age classes of *C. yacare* are located sequentially from negative to positive values, but there is a great overlapping among them. Therefore, shape changes associated to PC2 (Figs. 3C and 3D), among other components, seem related to the age gradient.

**Figure 4:**
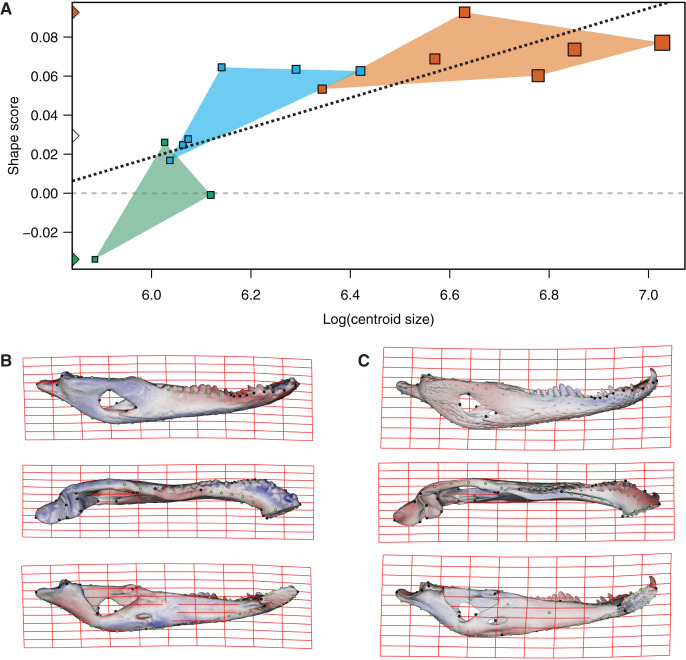
Analysis of multivariate regression of the Procrustes coordinates against the log-transformed centroid size for *C. latirostris*. (A) Shape scores *vs* log CS. (B) Thin plate spline gridlines and colored meshes of negative shape score exaggerated three times. (C) Thin plate spline gridlines and colored meshes of positive shape score exaggerated three times.

**Figure 5:**
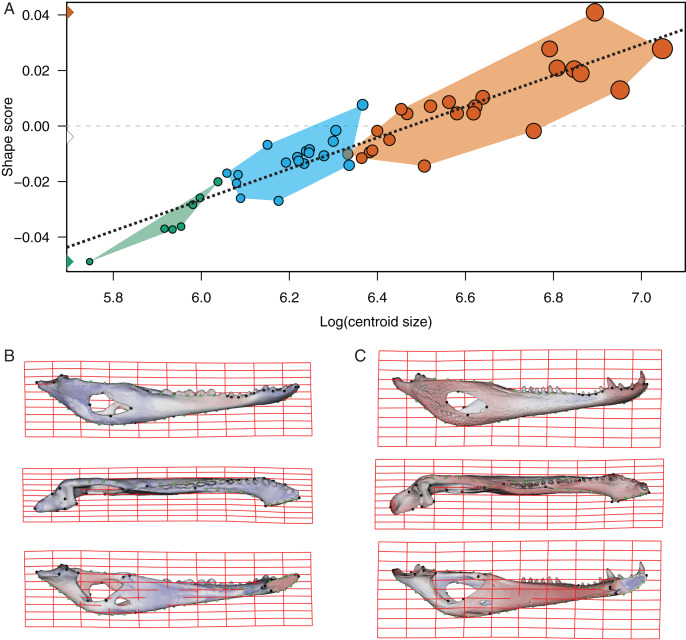
Analysis of multivariate regression of the Procrustes coordinates against the log-transformed centroid size for *C. yacare*. (A) Shape scores *vs* log CS. (B) Thin plate spline gridlines and colored meshes of negative shape score exaggerated six times. (C) Thin plate spline gridlines and colored meshes of positive shape score exaggerated six times.

In the multivariate regressions, a slightly common pattern of ontogenetic morphological change, shared by the two species, can be detected (Figs. 4 and 5). The angular comparison between these two regression vectors (in their own shape space) was 65.368° (*p*-value < 0.0001; *i.e*., non-orthogonal). In addition, these two regression vectors are orthogonal to all the PCs except PC1 and PC2. The angular comparison between these vectors (*i.e*., regressions and PCs) has a higher vector angle for PC1 (*i.e*., >72°) than PC2, which was 34.01° for *C. latirostris* and 50.71° for *C. yacare* (all *p*-values < 0.0001) which means that, although morphological changes of PC2 could be interpreted as a shared pattern of morphological change along ontogeny (see above), they resemble more the ontogeny of *C. latirostris* than *C. yacare* (see below). Morphological transformations from juveniles to adults can be observed in the thin plate spline gridlines. Allometry accounts for 19.01% in *C. yacare* and 22.31% in *C. latirostris* proportions of the total shape variation. The juveniles of both species have low and narrow mandibles with a small internal mandibular fenestra, a wide Meckelian fossa, a ventrally curved dorsal area of the surangular bone, a small and less curved (less concave) retroarticular process, a low and straight symphyseal area (the anterior portion is closer to the sagittal plane), procumbent teeth, and a straight postero-ventral area of the angular bone (Figs. 4B and 5B). Morphological changes towards adults show high and wide mandibles with a large internal mandibular fenestra, an elongated Meckelian fossa, a dorsal curved dorsal area of the surangular bone, a large and more curved (more concave) retroarticular process, a high and curved symphyseal area (the anterior portion is further to the sagittal plane), not procumbent teeth, and a curved postero-ventral area of the angular bone (Figs. 4C and 5C). In adults of *C. yacare* the symphyseal area becomes longer, narrower and more pointed. Although both caiman species share this pattern of morphological change along ontogeny, *C. latirostris* has higher Procrustes distances and lower centroid size amplitude between all specimens in the sample (*i.e*., 1.097 and 767.33, respectively) than *C. yacare* (*i.e*., 0.905 and 837.01; see Supplementary Information, Figs. 1 and 2). Consequently, some features are more evident (more colored) from early ontogenetic stages in *C. latirostris*. In addition, juveniles of *C. yacare* has slender mandibles with more procumbent teeth, lower symphyseal areas, high articular bones, larger external mandibular fenestrae, and a slender and more elongated Meckelian fossa than *C. latirostris*.

**Figure 6:**
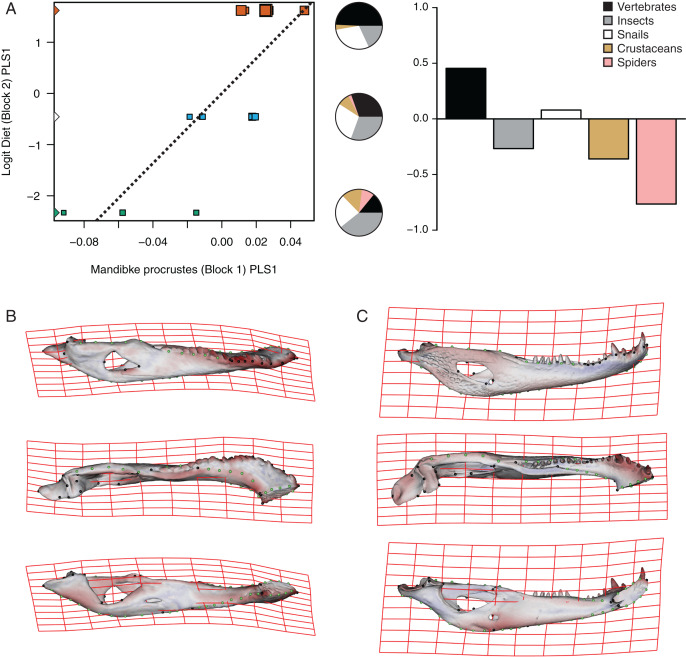
Analysis of PLS of the Procrustes coordinates (Block1) and Logit diet (Block2) for *C. latirostris*. (A) Scores of first pair of PLS. (B) Thin plate spline gridlines and colored meshes of negative shape score. (C) Thin plate spline gridlines and colored meshes of positive shape score. Colour intensity is proportional to the amount of change.

**Figure 7:**
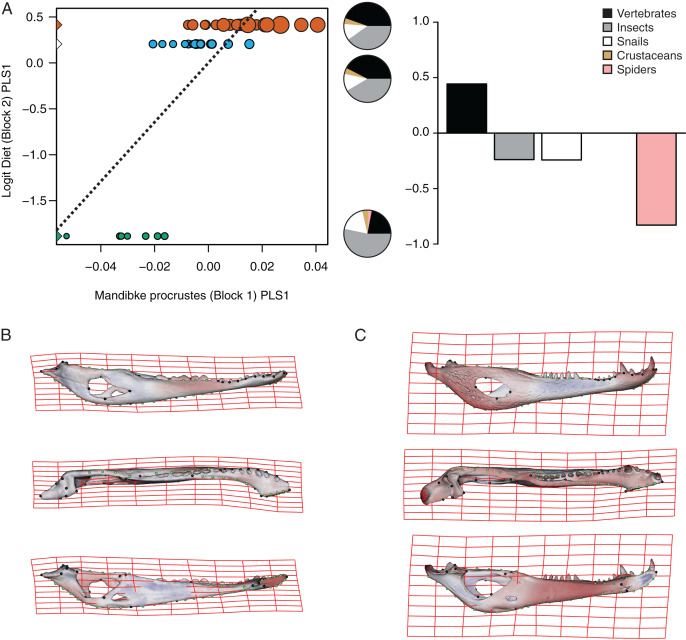
Analysis of PLS of the Procrustes coordinates (Block1) and Logit diet (Block2) for *C. yacare*. (A) Scores of first pair of PLS. (B) Thin plate spline gridlines and colored meshes of negative shape score. (C) Thin plate spline gridlines and colored meshes of positive shape score. Colour intensity is proportional to the amount of change.

The PLS analysis on both species (Figs. 6 and 7) show a significant relationship between shape and diet (Table 3). The PLS analysis on *C. latirostris* shows that the first pair of PLS explains about 98.41% of covariation. The block-2 PLS coefficients of the five diet categories segregate spiders, crustaceans and insects items to negative values (ca −0.766, −0.36 and −0.267 respectively), and vertebrates and snails items to positive values (~0.454 and 0.079 respectively) (Fig. 6A). The PLS1 scores show a high and significant correlation between blocks. While juveniles lie on the double negative quadrant (with highly negative values on block-2), sub-adults lie near the zero values (but slightly displaced on negative values of block-1) and adults are on the double positive quadrant.

**Table 3:** Partial least squares analyses for each species.

Species	Pair of axes	Singular value	% Total covar.	Pearson correlation coefficient	*p*-value
*C. latirostris*	PLS1	0.0444377	98.419	0.7983	0.0009
	PLS2	0.0056318	1.581	0.7135	0.1935
*C. yacare*	PLS1	0.0104369	94.857	0.7311	0.0001
	PLS2	0.0024302	5.143	0.577	0.1262

The PLS analysis on *C. yacare* shows that the first pair of PLS explains about 94.85% of covariation. The block-2 PLS coefficients of the five diet categories segregate spiders, snails and insects items (ca −0.831, −0.242 and −0.235, respectively) to negative values, and vertebrate items to positive values (~0.442) (Fig. 7A). The PLS1 scores show a high and significant correlation between blocks. While juveniles lie on the double negative quadrant (with highly negatives values on block-2), sub-adults lie near the zero values (but slightly displaced on negatives values of block-1) and adults are located on the double positive quadrant (but slightly displaced on negatives values of block-1).

Morphological changes of the PLS1 vector of block-1 in both species are visualized as surface plus thin plate spline gridline deformations in Figs. 6B, 6C, 7B and 7C. Transformations match with those of the regression analysis (see above) and show an angle between regression and PLS shape change vectors of 17.902° in *C. latirostris* and 19.599° in *C. yacare* (*p*-value < 0.00001; *i.e*., non-orthogonal).

## Discussion

### Ontogenetic mandibular transformations in *Caiman latirostris* and *Caiman yacare*

We have clearly identified a common pattern related to ontogenetic change in the mandible of both *Caiman* species (PC2; Figs. 3C and 3D). Juveniles have slender mandibles with a smaller internal mandibular fenestra (IMF), low and narrow symphyseal and post-dentary areas, and a less concave retroarticular process, whereas adults have higher and wider mandibles with a larger IMF, and more concave, longer and wider retroarticular processes. Besides, symphyseal teeth are splayed outward at early stages of both species, whereas common ontogenetic transformations imply a shift that results in relatively more robust mandibles with less procumbent teeth in adult specimens. Given the substantially little attention that crocodylian mandibles have received compared to cranium, and the limited conclusions offered by the literature in this regard, it is difficult to establish comparisons of our results with other crocodylians or even with the same species analyzed here. In this sense, the only two works that can be analyzed here are those of Monteiro & Soares (1997) and Verdade (2000), which have defined some cranial and mandibular linear measurements in specimens of *C. latirostris* and *C. yacare* and, in accordance with our findings, have detected that adult caimans have higher mandibles with longer retroarticular processes (Monteiro & Soares, 1997). No other meaningful comparison can be made with the available literature.

In this study, we could observe that some morphological features (*e.g*., retroarticular process inclination) remain unchangeable during the post-hatching life of the two caiman species. However, Fernandez Blanco (2019) has demonstrated that during the pre-hatching ontogeny of these two caiman species, the retroarticular process (still in its cartilaginous state) changes its inclination, being extremely ventrally inclined in early embryonic stages to less inclined (almost horizontal) in later stages. Moreover, differences in the inclination of the retroarticular process between post-hatching specimens of both species are already established from early post-hatching stages or even immediately before hatchling. Our results show that *C. yacare* have a retroarticular process more horizontally oriented compared to *C. latirostris*. In this regard, some mandibular transformations are more conspicuous from the beginning of the post-hatching development in *C. latirostris* than in *C. yacare*. This occurs due to the greater range of morphological variation (*i.e*., Procrustes distances amplitude) present in specimens of *C. latirostris* compared to *C. yacare*, which result in adult robust morphotypes with taller symphyseal areas, smaller external mandibular fenestrae, a massive articular region and less procumbent teeth. On the other side, *C. yacare* shows a smaller range of mandibular morphological variation (but higher centroid size range), and as a consequence, adult forms showed slender mandible morphotypes, especially at the symphyseal and post-dentary areas. These morphological changes and differences between *Caiman* species are likely related to developmental processes (*i.e*., heterochrony) as it was already suggested by other authors in some extinct crocodylian species (Erickson & Brochu, 1999; Godoy et al., 2018) and even in *C. latirostris* (Monteiro & Soares, 1997). When a heterochronic event occurs, the synchronization in the timing of shape development between species (and their ancestors) is lost, and the analysis of the ontogenetic trajectories requires considering the phylogenetic relationships (see Catalano, Segura & Vera Candioti, 2019 and references there in). So far, if changes in ontogenetic trajectories between these two *Caiman* species represents extensions, contractions, of shifts among many different changes, to hypothesize a neoteny or peramorphosis process (see Piras et al., 2011) needs to be evaluated in a macroevolutionary framework.

Comparing the ontogenetic shifts in the crania and mandibles of *C. latirostris* and *C. yacare*, the ranges of transformation of both structures are the opposite in each species. In the present study, we found a wider range of morphological change in the mandibles of *C. latirostris* than its cranium whereas Fernandez Blanco, Cassini & Bona (2018) observed a greater variation in the cranium of *C. yacare* in comparison to its mandible. This difference is likely considering that, while the cranium is a complex structure with multiple embryonic origins and a more intricate ontogenetic development, the lower jaw constitutes a morphofunctional unit whose primary role is to capture, manipulate and process materials during feeding (Anderson, 2009; Monteiro & Nogueira, 2010; Anderson et al., 2011). Although the palate, rostrum and braincase are also related to feeding innovations (Cleuren & De Vree, 2000; Pierce, Angielczyk & Rayfield, 2008), they are skull structures more fully associated with specialized sensory organs and nervous and pneumatic systems (*e.g*., olfactory bulbs, eyes, inner ear, encephalon and cranial nerves, paratympanic and paranasal systems) which, in turn, are associated with other multiple ecological factors *e.g*., Witmer & Ridgely, 2008; Stubbs et al., 2013; Dufeau & Witmer, 2015). In this sense, the mandible might suffer a general morphological change relatively independent from the general morphology of the rest of the skull (but see correlations with some particular regions as the pterygoid flanges, among others; Porro et al., 2011). Besides, lower jaws can be regarded as appropriate targets for morphofunctional analyses that may offer significant insight into the feeding ecology of species (*e.g*., Porro et al., 2011; Stubbs et al., 2013; Walmsley et al., 2013).

Common ontogenetic changes in caiman mandibles are probably mainly related to the shift in the diet that caimans and other crocodylians undergo during growth, in which juveniles feed mainly on insects or small vertebrates while adults predominantly consume vertebrates (Diefenbach, 1979; Hall & Portier, 1994; Melo, 2002; Erickson, Lappin & Vliet, 2003; Borteiro, 2005; Borteiro et al., 2008). Juveniles of *C. yacare* and *C. latirostris* eat mainly snails, spiders and insects (also crustaceans in *C. latirostris*) and change later in ontogeny to a diet based mainly on vertebrates (also snails are significant in diets of adults of *C. latirostris*). This common intake modification undoubtedly needs to bring about more and stronger muscles to support greater bite forces for the manipulation of bigger and stronger prey (it was also proposed that bite forces in crocodylians are controlled by body size; *e.g*., Erickson et al., 2012). Tendons and muscles in charge of the stabilization and movement of the crocodylian jaws are inserted in crests, margins and areas of the angular, surangular, articular and coronoid bones, and in the Meckel’s cartilage. In this study, we observed that these mandibular areas experience morphological transformations during the development of both caiman species (Iordansky, 1964; Schumacher, 1973; Holliday & Witmer, 2007; Bona & Desojo, 2011; Holliday et al., 2013). Thus, the larger and more curved retroarticular process, the more curved postero-ventral area of the angular bone, the elongation of the Meckelian fossa, the increase in size of the IMF, and the change in shape of the dorsal margin of the surangular bone are morpho-anatomical transformations present in adult caimans probably related to mechanical advantages associated with the modification of the diet during life. Accordingly, several areas of muscular attachment in the cranium of both caiman species (*e.g*., mandible adductor and depressor muscles) also change during growth (Fernandez Blanco, Cassini & Bona, 2018). As a result, since we identified a high correlation between mandibular and dietary changes throughout post-hatching ontogeny, we can assume these morphological changes as an adaptation of adult caimans to capture larger and/or more agile prey. Among these morpho-anatomical modifications, there is a conspicuous change in the symphyseal area and the inclination of teeth in both *Caiman* species. In adult specimens of both species, the symphyseal area is higher and curved, and the anterior teeth are less procumbent (Figs. 4 to 7). In addition, in *C. yacare* the symphysis becomes longer and the anterior sector of the dentary is narrower and more pointed compared to *C. latirostris*. Among crocodylians, the symphyseal length is variable and is strongly involved in the mechanical response of the mandible to shaking and twisting loads: short mandibular symphysis perform well for feeding upon large prey while elongate symphyses have structural constraints to big sizes of prey (Walmsley et al., 2013; Lessner et al., 2019). In this regard, although caimans are brevisymphyseal crocodylians, the slight enlargement of the symphyses in the ontogeny of *C. yacare* could be linked to the capture of small, agile, and aquatic vertebrates accordingly to its most piscivorous diet (contrary to *C. latirostris*) in adult specimens (see discussion below).

### Morphological mandibular disparity between *Caiman* species

Two clearly distinguishable morphotypes could be registered from this three-dimensional geometric morphometric analysis. On the one hand, specimens of *C. yacare* have slender jaws with large fenestrae, a narrow articular area, and a retroarticular process horizontally positioned. Besides, the splayed outward teeth are supported by a low and curved symphyseal area. On the other side, the morphotype of *C. latirostris* is characterized by robust mandibles with smaller fenestrae, a wider articular area, and a retroarticular process postero-ventrally inclined. In this latter species, the symphyseal area is higher and straight and teeth are less procumbent. Furthermore, these interspecific differences were found to be correlated to ontogenetic changes in diet, and could be related to the item’s food hardness. We have observed that snails are important components of the diet of juvenile specimens of *C. yacare*, and crustaceans for juveniles of *C. latirostris*. Moreover, the snail percentage increases during the ontogeny of *C. latirostris*, and these gastropods become important components of the diet of adult specimens of this caiman species (Fig. 6). Additionally, subadult and adult categories of *C. yacare* show more morphological and dietary similarities between them (more slender mandibular morphotypes and diets with low content of hard prey-piscivorous prey) than with juveniles of this species (Fig. 7). By contrast, juveniles, subadults, and adults of *C. latirostris* are well distant in the PLS analysis which means that food items and morphology are well distinct between each age category. A possible explanation for all these inter- and intraspecific dietary and mandibular morphological differences could be related to the rigidity and hardness of the animals consumed by each species during growth, such as the incorporation of animals with shells in the diet of *C. latirostris vs* the higher intake of softer items like spiders, insects or even small vertebrates (*e.g*., fishes) in adults of *C. yacare*. In this way, robust mandibles as seen in *C. latirostris* would be more suitable for crushing snails and crustaceans. Supporting this idea, Monteiro & Soares (1997) stated that the short and broad cranium and mandible of *C. latirostris* are more suitable for shell breaking than the long slender snouted *C. yacare*. Moreover, Piras et al. (2014) hold that morphological variation of the rostrum is linked to the maximum size of prey, so that short rostral shapes would be associated with durophagy and the handling of less agile but stout prey (Daniel & McHenry, 2001; McHenry et al., 2006). Another probably reason for the morphological disparity observed between *C. latirostris* and *C. yacare* may be related to the feeding prey capture method used by each species. It has always been proposed that cranial morphology in crocodylians is closely related to feeding behavior, diet, habitat and mechanical performance (*e.g*., Langston, 1973; Magnusson, Da Silva & Lima, 1987; Busbey, 1995; Monteiro, Cavalcanti & Sommer, 1997; McHenry et al., 2006; Pierce, Angielczyk & Rayfield, 2008; Piras et al., 2009; Stubbs et al., 2013; Walmsley et al., 2013). However, recent works (McCurry et al., 2017) suggest that the technique used by crocodylians to catch and process food would constitute the major evolutionary driver of the crocodylian skull morphology.

Morphological disparity of the lower jaw could be due to the differential use of the habitat by both caiman species as well (Fernandez Blanco, Cassini & Bona, 2018). Under this hypothesis, stout mandibles would be more suitable for animals living in superficial and fully vegetated waters (as in *C. latirostris*; see below) whereas skinny jaws seem to be better prepared for more aquatic species (such as *C. yacare*; see below). In Argentina, *C. latirostris* and *C. yacare* coincide in much of their geographic distribution (*e.g*., Poletta, 2011) but the use of habitat is different, especially when they live simultaneously in the same area (Larriera & Imhof, 2006). In this regard, Magnusson, Da Silva & Lima (1987) state that crocodylian species can modify their feeding according to the overlapping in their distribution. Such as the case of *Paleosuchus trigonatus* (Schneider, 1801) and *C. crocodilus* (Linnaeus, 1758) that feed on similar items when they occupy the same habitat but their diets are completely different if they do not coexist. As there are no studies on the feeding strategies of *C. yacare* and *C. latirostris* in overlapping areas, the relationship between habitat, diet and foraging modes need to be further explored in future analyses. However, it can be stated that *C. latirostris* lives in the fully vegetated surface of lentic aquatic ecosystems (Medem, 1983; Yanosky, 1990; Larriera & Imhof, 2006; Poletta, 2011), contrary to *C. yacare* which prefers more profound water courses with scarce plants (Larriera & Imhof, 2006). Accordingly, mandibles of *C. yacare* would offer less resistance and facilitate movements in the water (as elongated crocodylian snouts do; see Cleuren & De Vree, 2000) whereas the head (including lower jaws) of *C. latirostris* would be more appropriate for swimming and food searching in shallow environments completely vegetated (Borteiro et al., 2008). Moreover, some authors have proposed that some cranial features would be more suitable for species that live in certain types of habitats (*e.g*., Fernandez Blanco, Cassini & Bona (2018) and cites therein). Magnusson, Da Silva & Lima (1987) affirm that, in general terms, the variety of cranial shapes in crocodylians would be related to habitat, and species with low and wide heads inhabit swampy areas while species with long and thin snouts develop in riverine habitats. In addition, Mertens (1943) has also suggested that some cranial features in *C. latirostris* would be more beneficial for the type of environments in which this species lives (Iordansky, 1973). Finally, it is worth mentioning that the implication of the phylogenetic component in the evolution of the cranial shape in crocodylians has been explored by Piras et al. (2014) and they have concluded that cranial shape in Alligatoridae is biased by phylogeny more than ecological conditions.

## Conclusion

This is the first three-dimensional geometric morphometric study that quantifies the morphological variation in the mandibles of caimans along the ontogeny. Each caiman species show a clearly distinguishable morphotype and both species exhibit a common pattern of ontogenetic change in the lower jaw. When comparing the range of morphological variation along ontogeny (as Procrustes distances amplitude) in both species, we observe that this spectrum is greater in *C. latirostris*, and some mandibular transformations are more conspicuous from the beginning of the post-hatching development of this species. As a consequence, grown specimens of *C. yacare* retain some juvenile mandibular features, resulting in a slender morphotype, depicting a probable case of heterochrony.

Common ontogenetic morphological changes in caiman mandibles may probably be related to the common shift in the diet that crocodylians undergo during growth, whereas interespecific differences could be related to item food hardness and the variation in the percentage of each item consumed by each species during different ontogenetic stages. Furthermore, we propose that part of the lower jaw morphological disparity may be related to the differential use of the habitat by both caiman species as well. However, all these hypotheses should be tested through a morpho-functional approach. Although there are several works on the biomechanical and hydrodynamic properties of the crocodylian skull, further work is needed on mandibles. In this sense, a study of the associated morphological changes of the cranium and mandible in crocodylians would be relevant to achieve a complete understanding of craniomandibular mechanics and how it operates throughout the complete ontogeny.

## Supplemental Information

10.7717/peerj.15548/supp-1 Supplemental Information 1 Boxplots for *Caiman latirostris* showing differences in centroid size and differences in Procrustes Distances differences among the three post-hatching age categories.

10.7717/peerj.15548/supp-2 Supplemental Information 2 Boxplots for *Caiman yacare* showing differences in centroid size and differences in Procrustes Distances differences among the three post-hatching age categories.

## Additional Information and Declarations

### Data Availability

The following information was supplied regarding data availability: The data is available at the CONICET Digital Repository: Fernandez Blanco, María Victoria; Cassini, Guillermo Hernán; (2023): *Caiman yacare*, *Caiman latirostris*; Mandibula; Configuraciones de landmarks; ontogenia; modelos mandibulares tridimensionales. Consejo Nacional de Investigaciones Científicas y Técnicas. (dataset). http://hdl.handle.net/11336/184901.
