# Supplementary material for: A three-dimensional geometric morphometric analysis of the morphological transformation of Caiman lower jaw during post-hatching ontogeny
Source: PeerJ. 2023 Jul 12;11:e15548. doi: 10.7717/peerj.15548 (PMC10349558; doi:10.7717/peerj.15548)
Supplement: Supplemental Information 1 [file peerj-11-15548-s001.pdf]

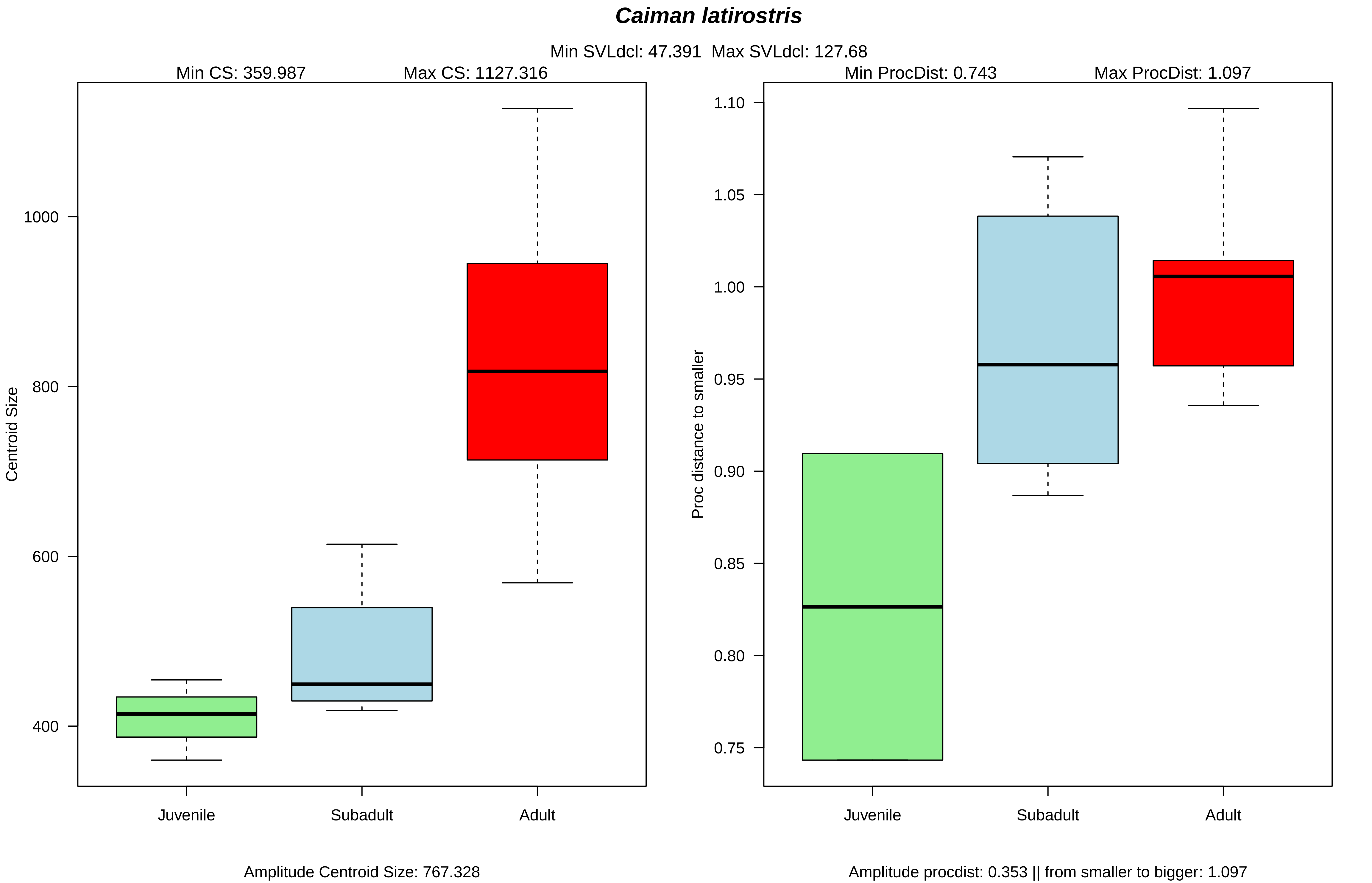

Caiman latirostris
Min SVLdcl: 47.391 Max SVLdcl: 127.68
Min CS: 359.987
Max CS: 1127.316
Centroid Size
1000
800
600
400
Juvenile
Subadult
Adult
Amplitude Centroid Size: 767.328
Min ProcDist: 0.743
Max ProcDist: 1.097
Proc distance to smaller
1.10
1.05
1.00
0.95
0.90
0.85
0.80
0.75
Juvenile
Subadult
Adult
Amplitude procdist: 0.353 || from smaller to bigger: 1.097
