# Supplementary figures and images for: A three-dimensional geometric morphometric analysis of the morphological transformation of Caiman lower jaw during post-hatching ontogeny
Source: PeerJ. 2023 Jul 12;11:e15548. doi: 10.7717/peerj.15548 (PMC10349558; doi:10.7717/peerj.15548)

# *Caiman yacare*

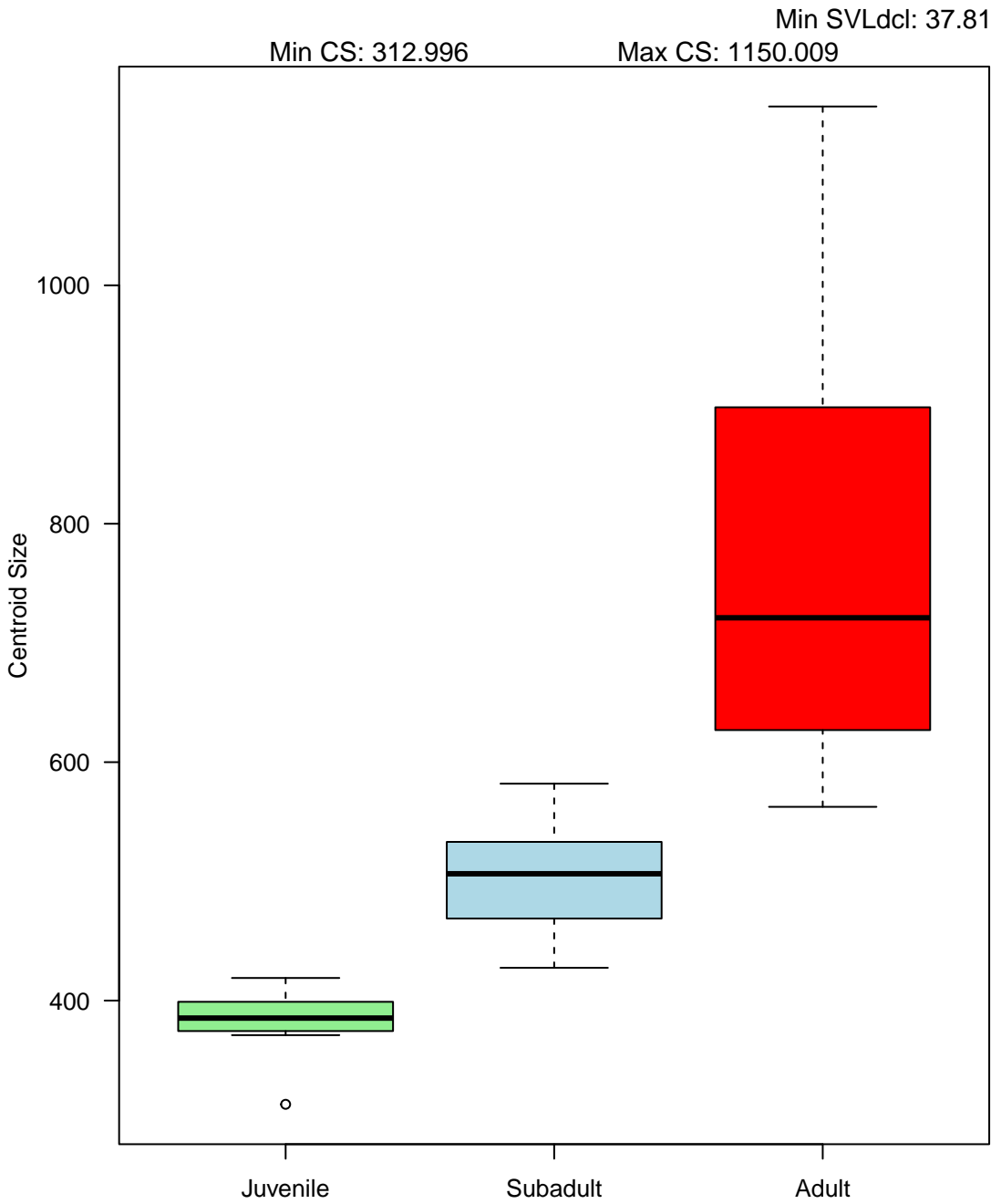

Amplitude Centroid Size: 837.012

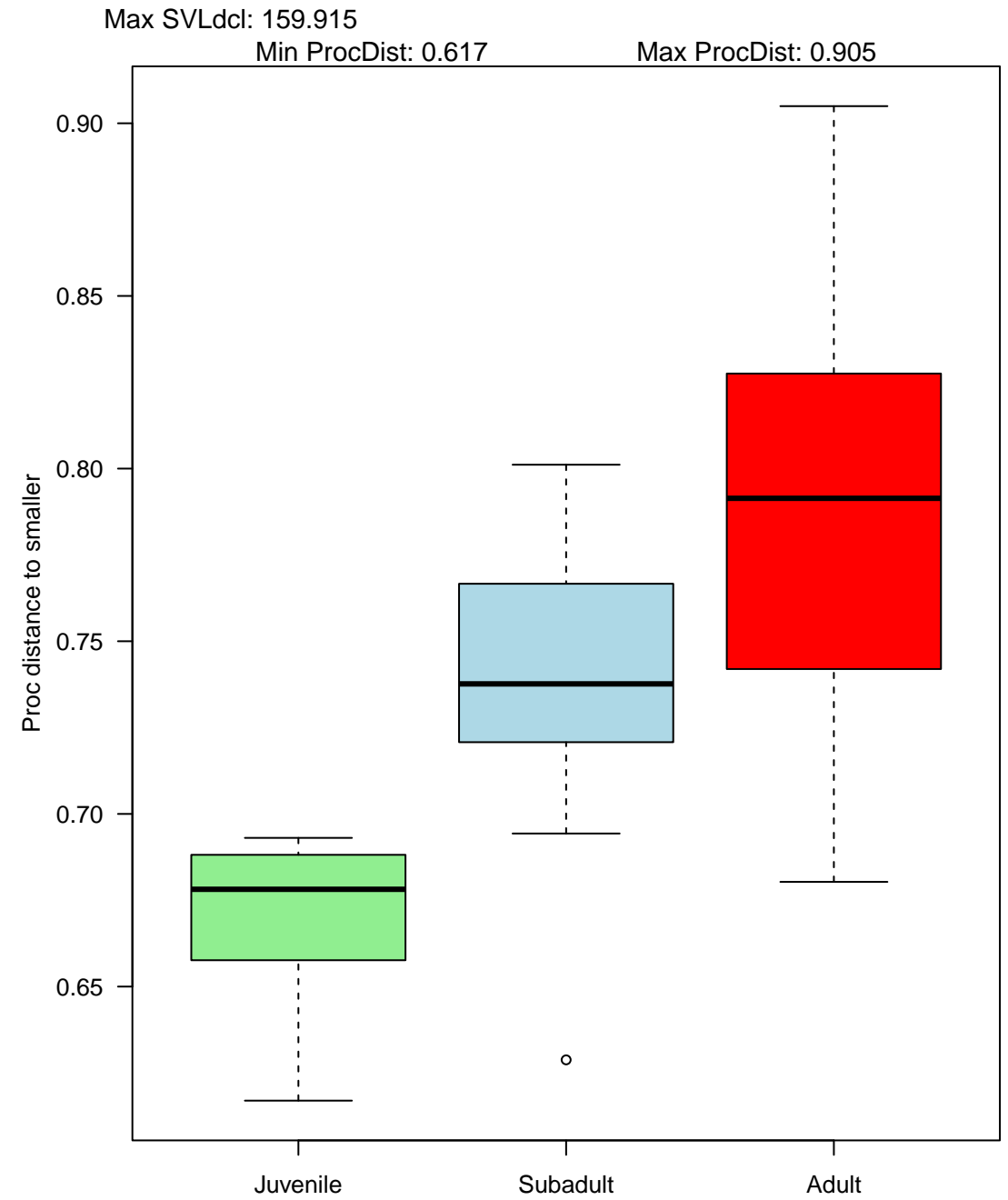

Amplitude procdist: 0.288 || from smaller to bigger: 0.905

Supplement: Supplemental Information 2 [file peerj-11-15548-s002.pdf]
